# Visual read performance of ^18^F‐Florbetapir and ^18^F‐NAV4694 Aβ PET compared against Centiloid reference standard in a paired cohort

**DOI:** 10.1002/dad2.70426

**Published:** 2026-07-16

**Authors:** Ishara Paranawithana, Vincent Doré, Pierrick Bourgeat, Aurora Poon, H. B. Toh, Tanyaluck Thientunyakit, Tawika Kaewchur, Antony Sutherland, Ashley G. Gillman, Kun Huang, Azadeh Feizpour, Jurgen Fripp, Victor L. Villemagne, Christopher C. Rowe

**Affiliations:** ^1^ The Australian e‐Health Research Centre Commonwealth Scientific and Industrial Research Organization Melbourne Victoria Australia; ^2^ Department of Molecular Imaging & Therapy Austin Health Melbourne Victoria Australia; ^3^ The Australian e‐Health Research Centre Commonwealth Scientific and Industrial Research Organization Brisbane Queensland Australia; ^4^ Department of Nuclear Medicine The Royal Melbourne Hospital Parkville Victoria Australia; ^5^ Faculty of Medicine, Siriraj Hospital Mahidol University Bangkok Thailand; ^6^ PET/CT and Cyclotron Center, Faculty of Medicine Chiang Mai University Chiang Mai Thailand; ^7^ The Florey Institute of Neuroscience and Mental Health The University of Melbourne Parkville Victoria Australia; ^8^ Department of Psychiatry University of Pittsburgh Pittsburgh Pennsylvania USA

**Keywords:** Alzheimer's disease, amyloid imaging, centiloid quantification, florbetapir, NAV4694, positron emission tomography, visual reads

## Abstract

**INTRODUCTION:**

Visual assessment remains standard practice to rule out amyloid‐β (Aβ) pathology. ^18^F‐NAV4694 (NAV) has high affinity for Aβ potentially detecting lower levels than other F‐18 Aβ tracers.

**METHODS:**

One hundred fifty participants in the AIBL study underwent both ^18^F‐Florbetapir (FBP) and NAV Aβ PET scans. PET scans were assessed by six nuclear medicine physicians against Centiloid (CL) quantification. An optimized reference region was utilized for FBP as it improved CL–visual read correlations. Inter‐reader agreement of visual assessment was measured using Fleiss’ Kappa.

**RESULTS:**

Mean peak accuracy for visual read exceeded 95% for both NAV and FBP. However, peak accuracy for NAV visual reads was achieved at 14–15CL compared to 38–46CL for FBP. Higher inter‐reader agreement was observed for NAV compared to FBP.

**DISCUSSION:**

For mild to moderate elevation in Aβ, visual read of NAV is more sensitive and consistent than visual read of FBP by both experienced and novice readers.

## Highlights

- NAV PET visual reads are more sensitive and consistent than FBP in low to moderate Aβ loads.
- Expert and novice readers show comparable performance with NAV at similar CL thresholds.
- Adding hemispheric white matter to FBP reference region improves CL match to visual reads and NAV CL.

## 1 BACKGROUND

Amyloid‐β (Aβ) positron emission tomography (PET) imaging biomarkers provide non‐invasive in‐vivo identification of Aβ plaques in the brain, allowing more accurate diagnosis of Alzheimer's disease (AD). Aβ PET is also being used for selection of eligible candidates and to track outcomes of Aβ targeting disease‐modifying treatments.^1^, ^2^, ^3^ Three ^18^F‐labeled Aβ tracers, Florbetapir (FBP), Florbetaben (FBB), and Flutemetamol, have been approved by the U.S. Food and Drug Administration (FDA) and European Medicines Agency for clinical use.^4^, ^5^, ^6^ In current clinical practice, visual assessment plays a key role in identifying individuals with abnormal Aβ pathology.^7^, ^8^ However, those ^18^F‐labeled Aβ tracers have relatively high non‐specific binding which could complicate visual interpretation of PET scans, particularly in individuals with low Aβ load.^9^ ^18^F‐NAV4694 (NAV) is an Aβ tracer that has higher target to background ratio,^10^ potentially making it a promising tool for diagnosis, management and post‐treatment monitoring of AD.

The Centiloid (CL) quantification is a standardized universal framework developed to estimate brain Aβ burden,^11^ providing a way to compare imaging results across different tracers and processing pipelines in multi‐center studies.^12^, ^13^, ^14^ Due to its robustness and interpretability, CL thresholds have been used to define secondary end points in interventional clinical trials,^3^, ^15^ as well as a completion criteria to decide when to discontinue treatment^1^. Several studies, including the IDEAS and AMYPAD studies which consisted of a large number of FBP scans among the other FDA‐approved Aβ tracers have reported strong agreement between visual assessment and CL quantification.^7^, ^12^, ^16^

Although quantification can be used to determine the presence of Aβ pathology, visual read of PET scans remains the current standard in clinical practice.^14^, ^17^ However, the outcome of visual assessment can vary depending on several factors, including the tracer used and the level of reader experience.^7^, ^8^, ^17^ Additionally, motion artifacts, poor scan quality, severe brain atrophy, and other abnormalities may further complicate visual interpretation for readers.^8^ Previous reports indicate that a CL value above 20 generally corresponds to visual positivity, reflecting moderate‐to‐frequent Aβ plaques.^14^, ^18^, ^19^, ^20^ More recent studies suggest a cutoff of 17 CL for highly experienced readers^21^ and 40 CL for readers in general memory clinic settings.^22^ However, these studies were mainly limited to FDA approved PET tracers.^16^, ^17^ The effect of reader experience on visual assessment performance across different Aβ tracers in paired cohort remains poorly understood.

The main aim of this study was to compare the performance of visual read between FBP and NAV Aβ PET scans against CL quantification as the reference standard, using a cross‐sectional sample of participants representative of a memory clinic setting. We used Fleiss’ Kappa score to evaluate inter‐reader agreement and reliability of visual assessment across six readers and performance metrics of accuracy, sensitivity, and specificity to measure concordance between quantification and visual read. A range of thresholds between 10 and 70CL were considered to assess the visual read performance at different positivity thresholds for each tracer. To test the effect of readers’ prior experience with Aβ PET scans on visual assessment outcomes, we further compared the performance between experienced and novice readers for the two Aβ PET tracers considered in this study.

## 2 METHODS

### 2.1 Participants

One hundred and fifty participants in the Australian Imaging Biomarkers and Lifestyle (AIBL) study of aging^19^, ^23^ who underwent an FBP and NAV Aβ PET scans within one year if cognitively impaired and two years if cognitively unimpaired were included in this study. All participants completed neuropsychological assessments, including the Mini‐Mental State Examination (MMSE) and Clinical Dementia Rating Scale Sum of Boxes (CDR‐SoB), to confirm diagnoses of AD, mild cognitive impairment (MCI), or cognitively unimpaired (CU). A blinded clinician panel provided clinical diagnoses. Participants were over 51 years, fluent in English, and had no history of neurological, psychiatric, or substance‐related disorders.

### 2.2 Study design

Participants were selected from the AIBL study,^19^, ^23^ with a near‐equal split of Aβ+ and Aβ− scans based on 20CL threshold to reflect a typical real‐world memory clinic population. Deidentified NAV and FBP PET scans were visually assessed by six board certified nuclear medicine physicians, three experienced (C.C.R., A.P., H.B.T.) and three novices (T.T., T.K., A.S.), and compared against CL values, considering Aβ quantification as the reference standard. All readers were blinded to quantification and clinical diagnosis. Experts had over 20 years of experience in nuclear medicine, with at least 1 year of Aβ PET reading experience and novices had had no prior experience. All readers received NAV visual assessment guidelines developed by an expert (C.C.R.) and completed Avid Radiopharmaceuticals’ online training for FBP. NAV scans were read using a rainbow scale in MedImage/MedView (v12) individually adjusted so that the maximal color level was just becoming visible in some areas of the cerebellar white matter; FBP scans were read in inverted grayscale as per manufacturer recommendations.^24^ PET scans were classified as Aβ+ or Aβ− based on tracer uptake in gray matter regions: frontal cortex, posterior cingulate, precuneus, lateral temporal and parietal cortices, and striatum. Readers assigned scans to four categories: negative, probably negative, probably positive, or positive. Negative indicated no gray matter binding; probably negative, binding in one or two regions less than white matter; probably positive, binding equal to white matter in two or more regions; positive, binding higher than white matter in multiple regions.

### 2.3 PET image acquisition

^18^F‐NAV4694 and ^18^F‐Florbetapir were radiolabeled at the Department of Molecular Imaging and Therapy, Austin Health, Melbourne.^9^, ^19^ PET images were acquired 50 minutes post‐injection (200MBq ± 10% for NAV; 370MBq ± 10% for FBP) with 20‐minute static scan for each tracer. Acquisition was performed on Philips Gemini (NAV: 103; FBP: 61) and Allegro (NAV: 47; FBP: 4) scanners at Austin Health, and Siemens Biograph mCT (FBP: 85) at the Brain Research Institute in Melbourne. Images were reconstructed using row‐action maximum‐likelihood algorithm (RAMLA) for Philips scanners and ordered subset expectation maximization (OSEM) for Siemens.

**RESEARCH IN CONTEXT**

**Systematic review**: The authors reviewed the literature using PubMed, conference abstracts, and presentations. Visual assessment of amyloid‐β (Aβ) positron emission tomography (PET) scans remains standard practice to rule out Aβ pathology. However, accuracy and reliability of visual interpretation vary across ^18^F‐labeled tracers and depend on reader experience. No prior studies have compared visual read performance of NAV4694 with U.S. Food and Drug Administration (FDA)‐approved Florbetapir tracer.

**Interpretation**: NAV visual reads achieved overall peak accuracy at a substantially lower positivity threshold of 15 Centiloid (CL) [95% confidence interval [CI]: 8–22CL] compared to 46CL [24–50CL] for FBP. Our findings support the use of Aβ NAV for detection of low levels of cerebral Aβ plaques more accurately than FBP, aiding the visual exclusion of Aβ pathology.

**Future directions**: Larger head‐to‐head studies, harmonization strategies to address scanner variability, and validation against *post mortem* histopathology standard of truth are essential to confirm these results and establish standardized visual interpretation protocols.

### 2.4 Image analysis and quantification

All Aβ PET scans were processed using the CapAIBL PET‐only pipeline, with spatial normalization to a standard template via an adaptive atlas approach previously described.^25^ PET images were smoothed to a uniform 8 mm FWHM resolution, using scanner‐specific point spread functions estimated from a Hoffman phantom. Smoothing to a uniform resolution has been shown to improve quantification reliability across scanners and partially reduce longitudinal variability.^13^, ^26^ All normalized scans were visually inspected to confirm accurate registration.

Aβ load was quantified using standard CL masks in normalized space. NAV scans were scaled using the whole cerebellum as the reference region, while FBP scans used a composite reference of subcortical white matter and whole cerebellum.^13^, ^27^ This composite reference for FBP has been validated against large Pittsburgh Compound B (PiB) dataset from AIBL, Alzheimer's Disease Neuroimaging Initiative (ADNI), and Open Access Series of Imaging Studies (OASIS), with a new regression equation introduced for CL computation.^13^ As shown in the supplementary materials, using the composite reference region for FBP reduced variability, particularly at lower CL values, enhancing inter‐tracer agreement and correlation with visual reads.

### 2.5 Statistical analysis

To assess agreement in Aβ quantification, CL values from NAV and FBP scans were compared using the coefficient of determination (R^2^). Inter‐reader agreement for visual reads was evaluated using Fleiss’ Kappa across the full CL range. For this analysis, data were binned in 10CL intervals while retaining similar sample distributions between tracers. Visual read accuracy within each bin was assessed using the widely accepted 20CL threshold for Aβ positivity. Mean accuracy across readers was used to evaluate reliability along with inter‐reader agreement on visual read outcome.

Accuracy, sensitivity, and specificity were calculated by comparing visual reads against CL values as the reference standard. The primary analysis included all six readers to compare tracer performance. A secondary analysis grouped readers by experience to assess the impact of their prior experience on visual read performance. Metrics were evaluated across tracers and reader groups at thresholds ranging from 10 to 70CL. The CL threshold yielding the highest overall accuracy was considered optimal for each tracer. Confidence intervals for optimal thresholds were generated using 1000 bootstrap iterations with replacement, and 95% confidence intervals (CIs) were computed via the percentile method. Sensitivity and specificity at the optimal threshold were reported for both primary and secondary analyses as mean values with 95% CIs (in brackets), unless otherwise specified.

## 3 RESULTS

### 3.1 Participant demographics and clinical characteristics

Of the 150 participants, 88 (58.7%) were cognitively unimpaired (57 with subjective cognitive concerns, 31 without self‐reported memory concerns), 40 (26.7%) had mild cognitive impairment (MCI), and 22 (14.7%) had dementia due to AD (Table S1). AD individuals were younger and had more females (66.6%) than both CU and subjects with MCI. AD group showed the highest CDR‐SoB and lowest MMSE scores, significantly differing from CU and MCI. Apolipoprotein E (APOE) ε4 carriership was lowest in CU (28.7%) and highest in MCI (84.6%). Aβ+ and Aβ− scans were nearly balanced in the dataset (FBP: 76+/74−; NAV: 79+/71−) based on 20CL threshold. Ninety‐one (60.7%) participants underwent their FBP scan first. AD patients had shorter intervals between scans (6.4 months) than CU (21 months) and MCI (9.9 months).

### 3.2 Validation of CL quantification

**FIGURE 1:**
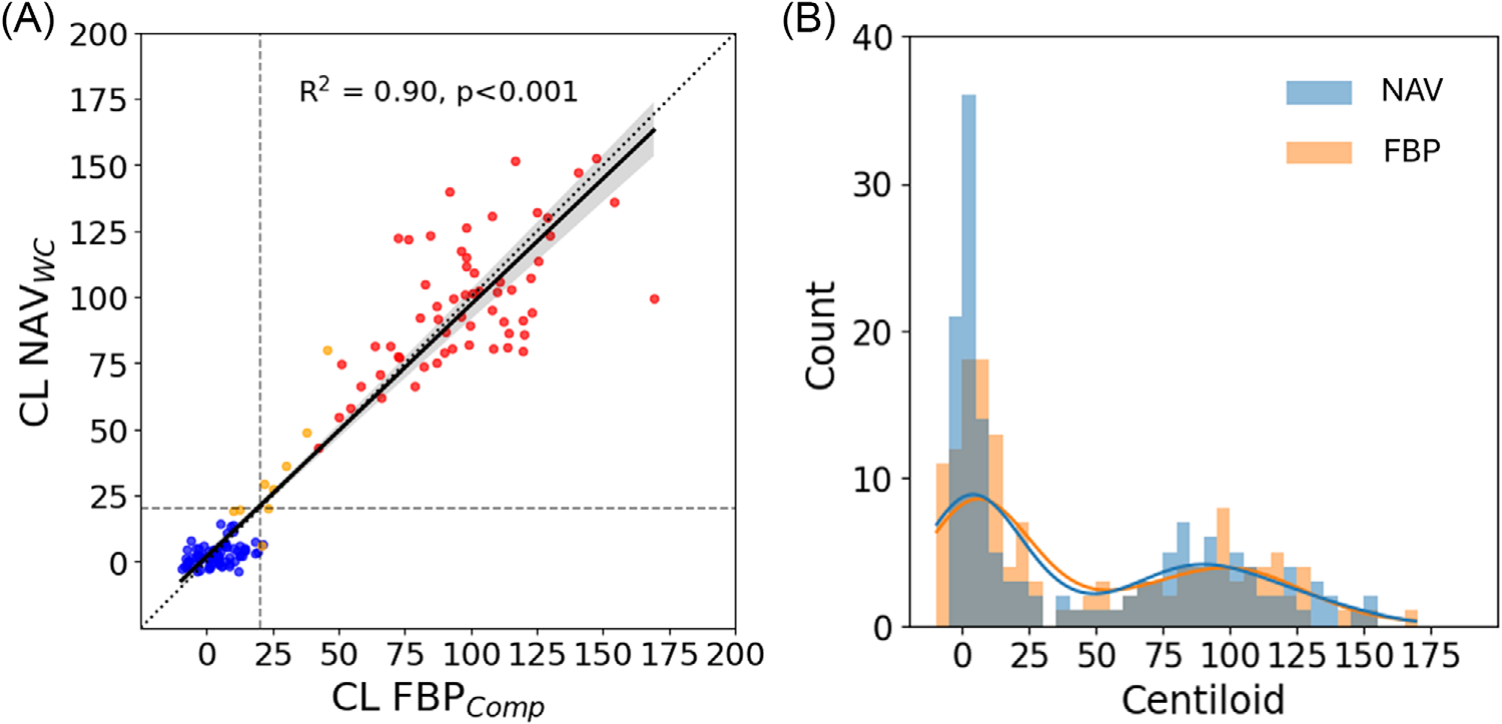
Agreement of Aβ quantification between the two PET tracers. (A) Scatter plot for NAV against FBP Centiloids with WC and composite region used as reference, respectively. Datapoints are color‐coded based on the majority vote of visual readers (≥ 4) with both NAV and FBP positive in red, both NAV and FBP negative in blue and NAV positive, FBP negative in yellow. (B) Histograms illustrate Centiloid distributions of FBP and NAV. Aβ, amyloid‐β; Comp, composite white matter; FBP, ^18^F‐Florbetapir; NAV, ^18^F‐NAV4694; PET, positron emission tomography; WC, whole cerebellum

We performed linear regression to assess inter‐tracer agreement and validate the use of CL quantification as the reference standard for visual assessment. Figure 1(A) shows a scatter plot comparing NAV (whole cerebellum reference) and FBP (composite white matter reference) CL values. Data points are color‐coded by majority visual read with both negative in blue, both positive in red, and NAV positive/FBP negative in yellow. There were no NAV negative/FBP positive cases. A strong correlation was observed between NAV and FBP CL (*R^2^* = 0.90, *p* < 0.001). Figure 1B) presents histograms showing bimodal CL distributions for both tracers, with peaks near 0 and 100CL and closely aligned kernel density estimates, further supporting strong quantification agreement between tracers. A supplementary scatter plot (Figure S1) using whole cerebellum as the reference for both tracers showed weaker agreement (*R^2^* = 0.82, *p* < 0.001), consistent with prior studies.^13^ These results highlight the improved inter‐tracer consistency when using a composite reference region for FBP.

**FIGURE 2:**
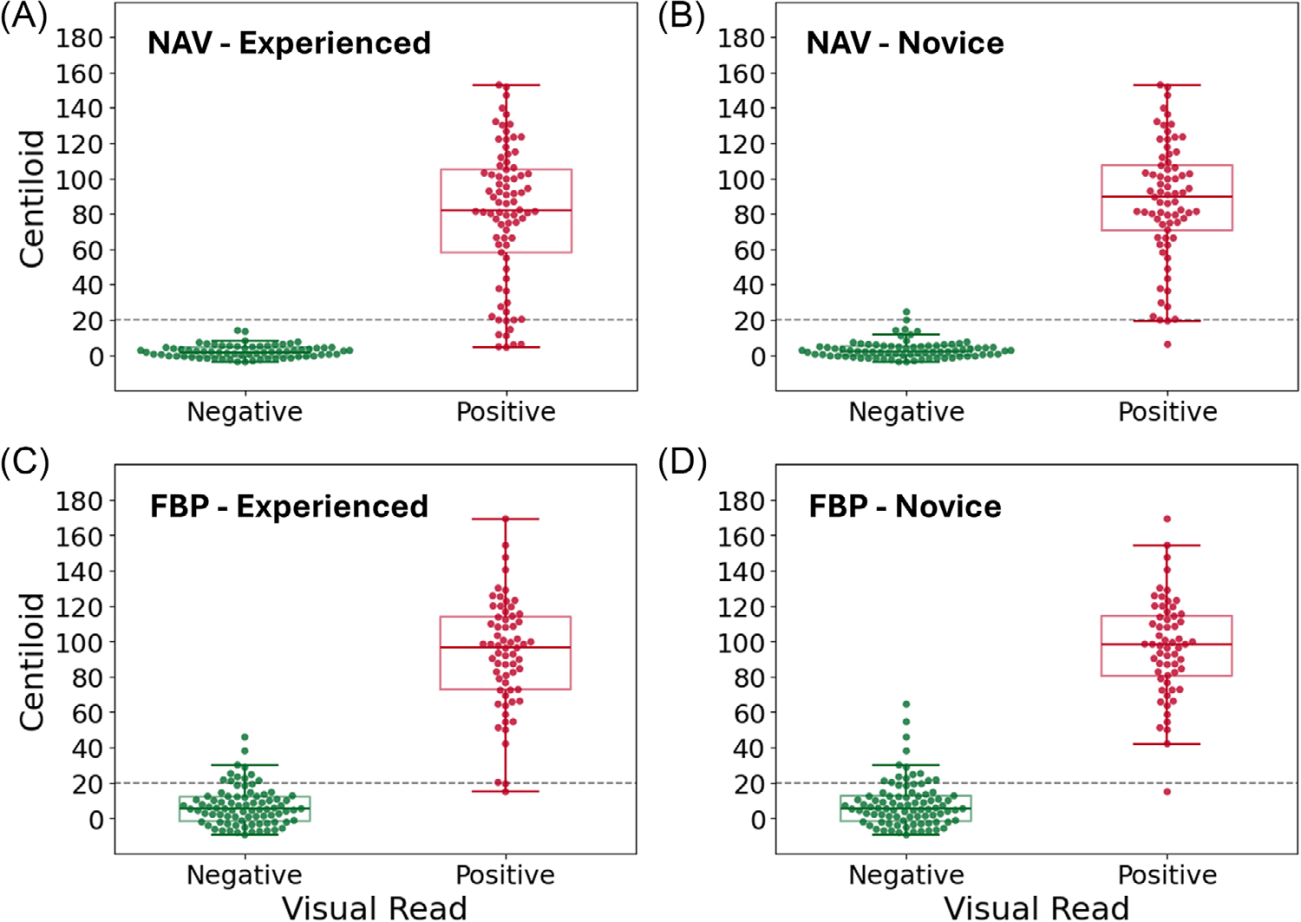
Swarm/box plots of CL quantification against the visual read based on the majority vote of readers. The top panels represent (A) experienced and (B) novice readers for NAV tracer. The bottom panels illustrate the results of (C) experienced and (D) novice readers for FBP tracer. The horizontal dashed line in each plot denotes the threshold of 20CL typically used to define Aβ PET positivity. Aβ, amyloid‐β; CL, Centiloid; FBP, ^18^F‐Florbetapir; NAV, ^18^F‐NAV4694; PET, positron emission tomography

**FIGURE 3:**
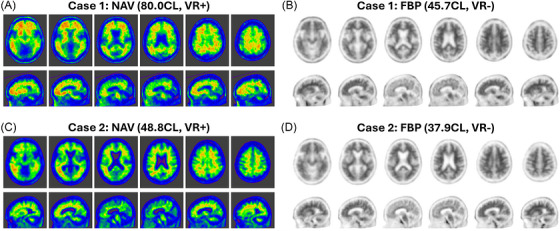
Aβ PET images of two discordant cases with NAV and FBP visual reads. All readers (three experts and three novices) read these scans as positive (VR+) with NAV and negative (VR–) with FBP. The estimated amyloid burden is presented for each scan along with visual read result. NAV images are shown in rainbow color scale (left), and FBP images are in inverted grayscale (right). Six transaxial and sagittal slices are presented as representative images for each tracer. For case 1, NAV scan shows tracer binding of moderate degree in frontal, precuneus/posterior cingulate, lateral parietal, and lateral temporal cortex and the striatum. The NAV scan of case 2 shows gray matter binding in the right lateral temporal and parietal cortex and right anterior striatum on transaxial images and mild binding in midline precuneus/posterior cingulate and orbitofrontal cortex on the mid sagittal image. In contrast, both FBP scans show delineation of white matter tracts throughout temporal and occipital areas and scalloped appearance of white matter in the frontal cortex which are typical visual features of a negative scan. Aβ, amyloid‐β; FBP, ^18^F‐Florbetapir; NAV, ^18^F‐NAV4694; PET, positron emission tomography; VR, visual read

Figure 2 shows visual assessment outcomes based on the majority vote of experienced (panels A and C) and novice readers (panels B and D) for NAV and FBP separately. When considering a generally accepted threshold of 20CL, both experienced and novice readers had good overall performance in correctly classifying scans as negative or positive with NAV (experienced: zero false negatives and 10 false positives, novice: one false negative and three false positives). In contrast to NAV, for FBP both experienced and novice readers read most scans in the low positive to moderate CL range (20‐50CL) as negative. Experienced readers had 11 false negatives and two false positive FBP reads while the novice readers had 14 false negatives and one false positive for FBP. However, nearly all positively read FBP scans presented much higher than 20CL. Representative PET images of two discordant cases where all readers had read the scan as positive (VR+) with NAV and negative (VR‐) with FBP are shown in Figure 3.

### 3.3 Inter‐reader agreement and reliability

**FIGURE 4:**
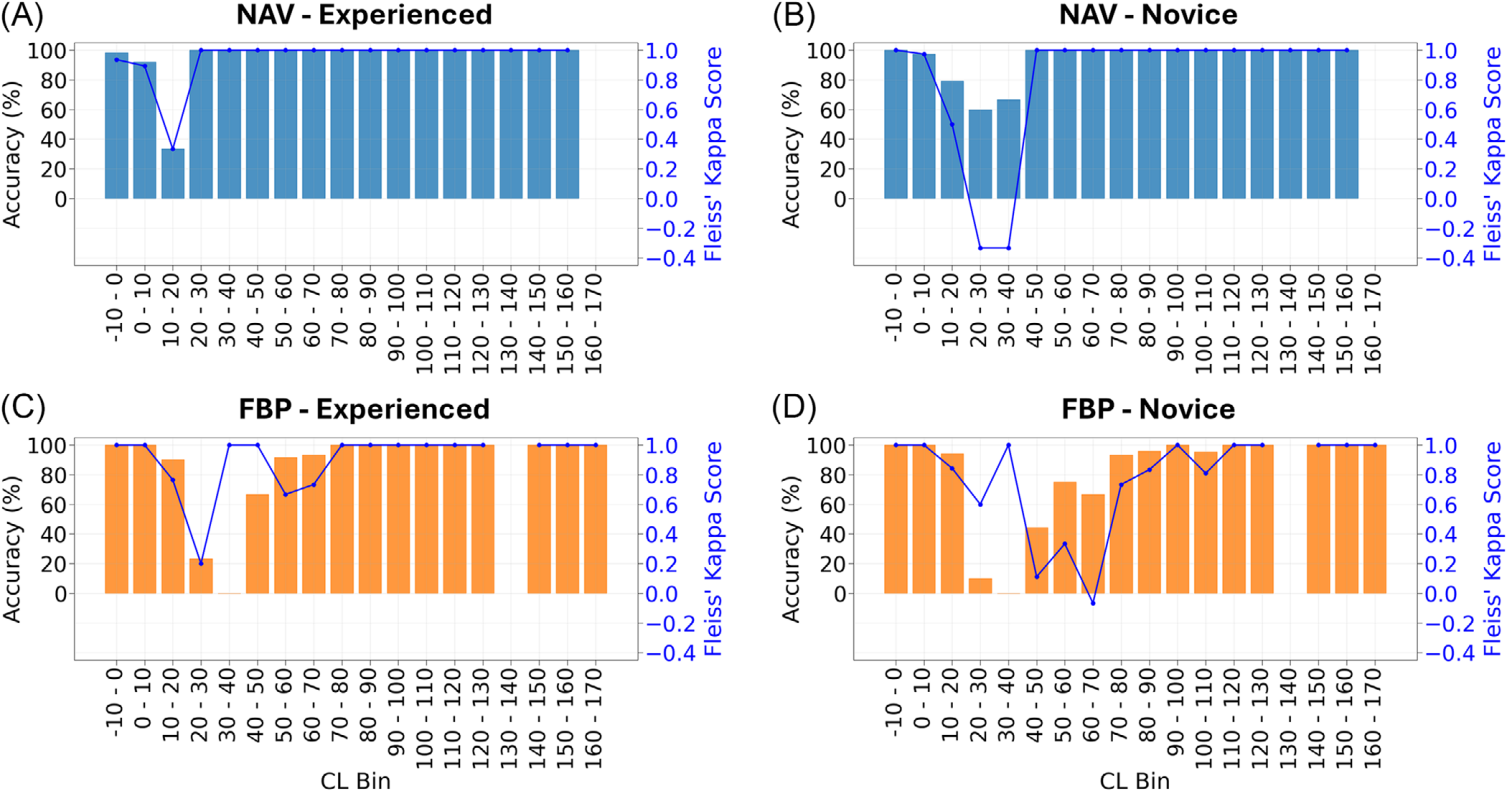
Mean accuracy of visual assessment (shown in bar charts) and Fleiss’ Kappa scores (shown in line plots) of experienced readers (left column, panels A and C) and novice readers (right column, panels B and D) for NAV and FBP in blue and orange, respectively. A Kappa score of 1 represents perfect agreement between readers whereas zero means no agreement beyond chance. Generally accepted threshold of 20CL was used for both tracers in the accuracy analysis. CL, Centiloid; FBP, ^18^F‐Florbetapir; NAV, ^18^F‐NAV4694

Fleiss’ Kappa and mean accuracy were used to assess inter‐reader agreement and reliability of visual assessments by experienced and novice readers. Experienced readers showed perfect agreement (Kappa = 1) and 100% accuracy for NAV scans above 20CL (Figure 4), with disagreement only between 10‐20CL. Novice readers performed less consistently, with a larger and deeper dip in Kappa and accuracy between 10 and 40CL. FBP showed greater variability in Kappa and accuracy across a broader CL range for both reader groups. Among novices, the FBP Kappa score ranged below 0.8 and accuracy below 80% between 20 and 70CL. A discrepancy between Kappa score and mean accuracy was noted in the 30–40CL bin for FBP, which included only one subject. All readers rated the FBP scan as negative (Kappa = 1), but the CL was 38, resulting in 0% accuracy. The NAV scan of the same subject had a CL of 49 and was rated positive by all readers. Figure 3C,D) shows transaxial and sagittal slices of NAV and FBP scans for this participant, with clear cortical uptake in the lateral temporal and parietal cortex (right more than left) and right striatum on the NAV scan. Inter‐reader agreement and accuracy results of combined readers are shown in Figure S2.

### 3.4 Comparison of visual assessment performance

**FIGURE 5:**
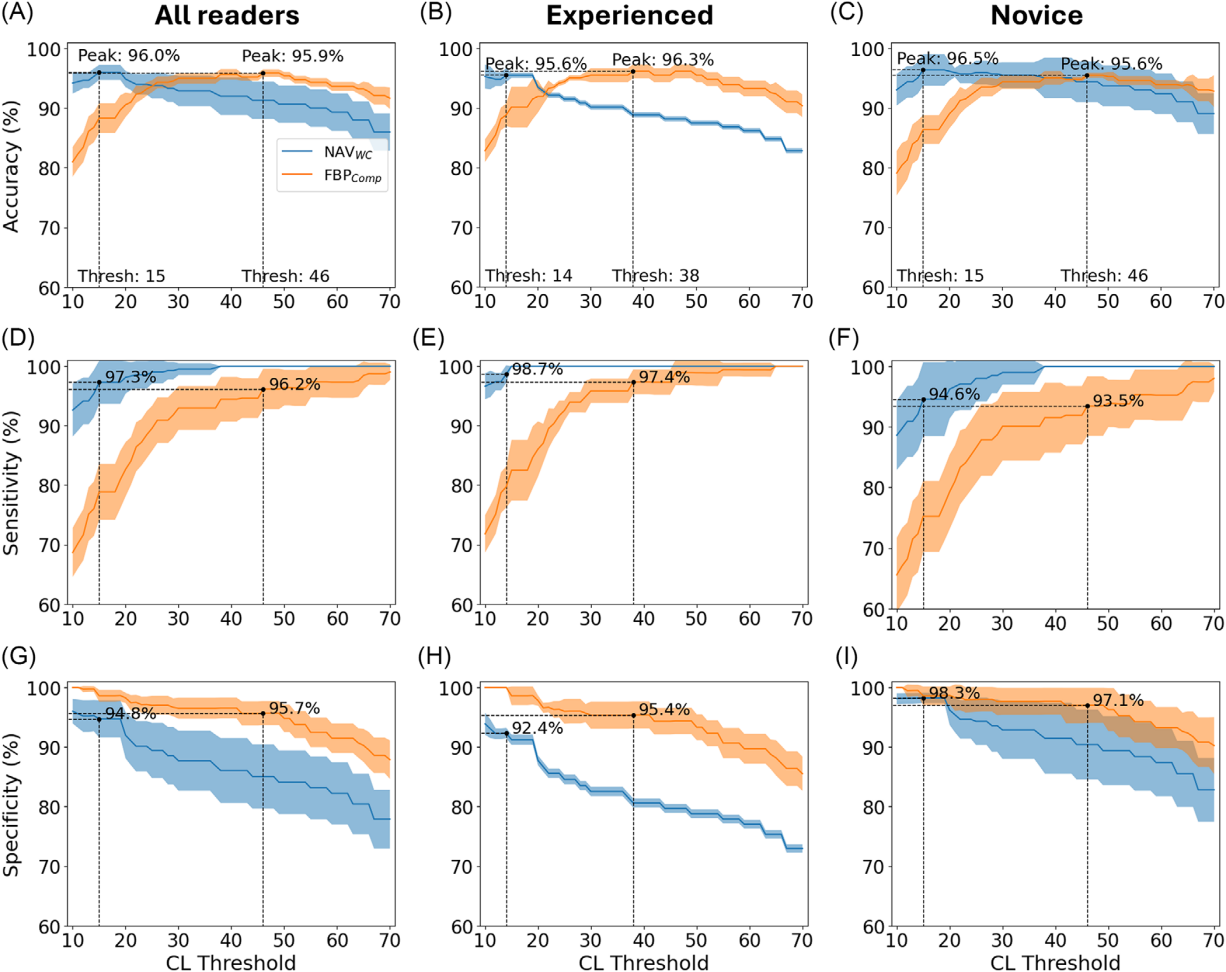
Performance metrics of visual assessment of Aβ PET scans against CL reference standard for different thresholds between 10 and 70CL. Comparison of accuracy (top row) between the two tracers. The left column represents combined results when all readers were considered. The results of experienced and novice readers are in the middle and right columns, respectively. The peak accuracy for each tracer and the corresponding threshold value are annotated in each panel in the top row. Error bars represent 95% CI across readers. Comparison of sensitivity (middle row) and specificity (bottom row) between the two tracers. Sensitivity and specificity at the optimal threshold (the threshold that produced the highest accuracy) are annotated for each case. Aβ, amyloid‐β; CI, confidence interval; CL, Centiloid; PET, positron emission tomography

To determine the optimal CL threshold for Aβ positivity in visual reads, a range of thresholds from 10 to 70CL were evaluated. When comparing visual assessments to CL reference standard, NAV achieved an overall mean peak accuracy of 96.0% [94.7–97.3%] at 15CL [8‐22CL], while FBP reached 95.9% [95.3–96.5%] at a higher threshold of 46CL [24–50CL] as shown in Figure 5. Novice readers showed similar results to experienced readers for NAV (peak accuracy 96.5% [93.8–99.1%] at 15CL [12–38CL] vs 95.6% [94.4–96.7%] at 14CL [8–15CL] respectively).

FBP peak accuracy was obtained at a higher CL threshold, 96.3% [95.1–97.4%] for experienced readers at 38CL [22–46CL], and 95.6% [95.1–96.0%] for novices at 46CL [26–50CL]. NAV maintained higher accuracy than FBP across its 95% CI threshold range, including the widely used 20CL cutoff. Figure S3 in the supplementary materials shows visual read accuracy across CL thresholds when whole cerebellum was used as the reference for both tracers. Results were consistent, with NAV achieving peak accuracy at a lower threshold than FBP.

Visual reader sensitivity and specificity at peak accuracy threshold for NAV and FBP are shown in Figure 5D‐I. For all readers, mean sensitivity was 97.3% [93.7–100.0%] for NAV and 96.2% [92.8–99.6%] for FBP. Mean specificity was 94.8% [91.6–97.9%] for NAV and 95.7% [93.7–97.7%] for FBP. Generally, NAV had higher sensitivity than FBP across the 10–70CL range but lower specificity consistent with better visual detection of low levels of Aβ. Although specificity was higher in novices compared to experienced readers, substantial variability was observed across thresholds. Overall, both tracers achieved above 90% sensitivity and specificity, with NAV performing better than FBP at lower CL thresholds.

**FIGURE 6:**
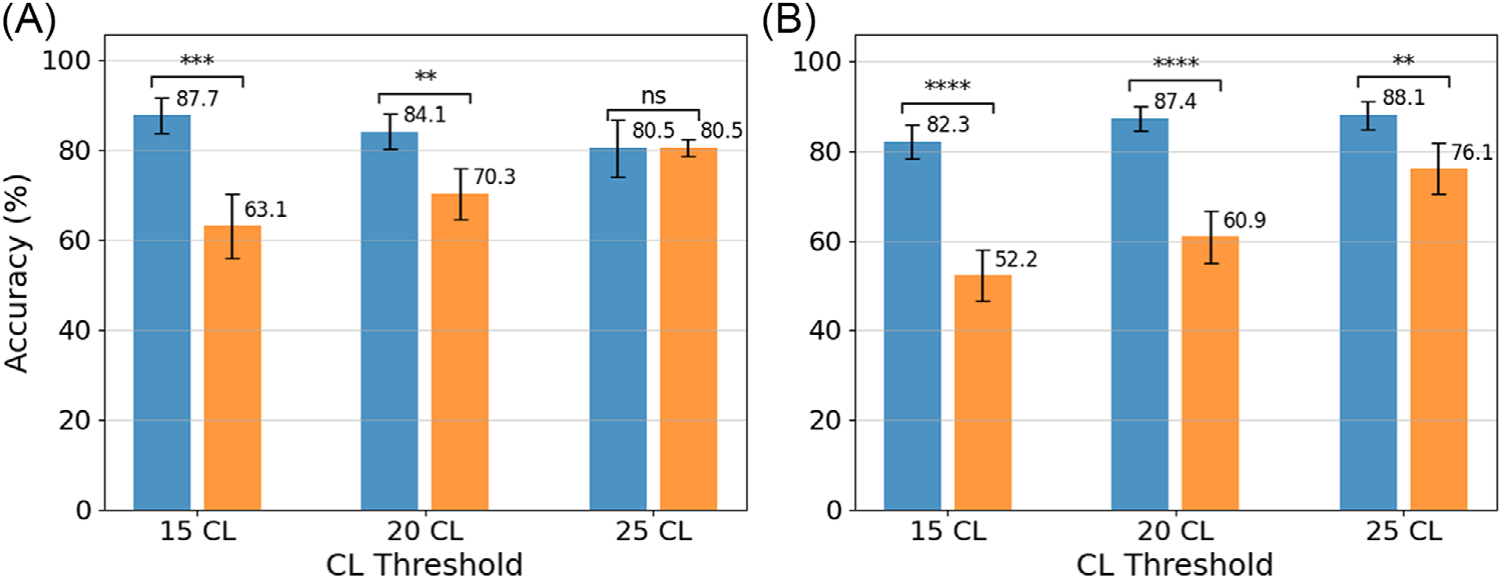
Visual assessment accuracy at three commonly used thresholds of 15, 20, and 25CL, only including data between 10 and 70CL of paired NAV‐FBP scans. (A) original analysis with “probably positive” and “positive” visual reads combined in the “positive” dichotomous group and (B) confidently positive only in the “positive” visual reads group and all other categories of visual read in the “negative” dichotomous group). 95% CI across all readers is shown in error bars and statistical significance comparing NAV (blue) to FBP (orange) is indicated by **p < 0.01, ***p < 0.001, and ****p < 0.0001. CI, confidence interval; CL, Centiloid; FBP, ^18^F‐Florbetapir; NAV, ^18^F‐NAV4694; ns, not significant

When assessing accuracy at three commonly used thresholds of 15, 20, and 25CL for paired NAV‐FBP scans where at least one scan was between 10 and 70CL (46 participants), NAV outperformed FBP at 15CL (87.7% vs 63.1%) and 20CL (84.1% vs 70.3%) (Figure 6A), with no difference at 25CL. Using confidently positive reads where only positive visual reads were retained in the positive group and all other visual categories classified as negative, NAV accuracy increased while FBP decreased: 15CL (NAV 82.3% vs FBP 52.2%), 20CL (87.4% vs 60.9%), and 25CL (88.1% vs 76.1%) (Figure 6B). This indicates that if aiming to read positive only above a 20‐25CL threshold, NAV is best read as positive only when binding in cortex visually exceeds white matter. In contrast, a similar restriction on FBP reduced accuracy in this range.

## 4 DISCUSSION

Fleiss’ Kappa indicated strong agreement for NAV, confirming reliable visual assessment across readers of varying experience. Novices performed worse than experienced readers, likely due to variable subjective interpretation of borderline cases. Both primary and secondary analyses showed tracer‐specific CL thresholds for optimal accuracy with NAV achieving peak performance at a lower threshold (14–15CL) than FBP (38–46CL). This pattern is consistent with findings for PiB, a tracer with similarly low non‑specific white matter binding^9^, which has been shown to require lower visual positivity thresholds than FBB.^28^ The higher threshold observed for FBP likely reflects greater and more variable white matter uptake, which degrades gray‐white matter contrast and increases discordance between visual reads and CL quantification, thereby requiring higher cortical signal before scans are confidently classified as visually positive.^17^, ^29^, ^30^

NAV thresholds were consistent across reader groups, whereas FBP thresholds varied, with novices needing a higher threshold, suggesting greater difficulty in identifying intermediate to moderate Aβ levels in FBP scans. Consistent with prior studies, novices demonstrated lower sensitivity for both tracers.^7^, ^8^ At the commonly used 20CL threshold, NAV showed higher sensitivity but lower specificity than FBP (Tables S2 and S3). These patterns align with recent findings comparing visual assessment and quantification across other Aβ PET tracers.^7^, ^12^ NAV accuracy improved at 20–25CL when only scans with cortical binding exceeding white matter were read as positive. Including two or more regions where cortical binding is equal to white matter increased accuracy at 15CL but reduced specificity at higher thresholds.

When investigating discordant visual reads based on the majority vote (i.e., different visual classification with NAV and FBP as shown in Figure S4), nine cases were read as positive with NAV and negative with FBP. Seven of these cases had a FBP CL level over 20. There was a larger time difference between scans for CU group (21.2 ± 3.3 months) compared to MCI and AD groups. However, most of those discordant cases (88.9% or 8 out of 9) have had their FBP scan first. On the contrary, no cases were visually read as positive with FBP and negative with NAV.

Most scans with CL values below ∼15CL were read as negative: 97.4% for NAV and 98.6% for FBP. In the borderline range of 15–25CL, where many published CL thresholds fall,^18^, ^21^ 100% of NAV scans (*n =* 6) were read as positive, compared to 0% for FBP (*n* = 11). Although this CL range is not strongly predictive of progression to MCI/AD over the next 7.5 years,^31^ it is associated with early Aβ accumulation^32^ and sparse neuritic plaques, as shown in *post mortem* studies.^18^, ^33^ Our results indicate that experienced readers can detect these very early changes with NAV but not with FBP (100% vs 18.2%). This enhanced sensitivity will have implications if using visual read to select persons for anti‐Aβ treatments or clinical trials. This illustrates the need for CL quantitation rather than visual reads for selection to ensure consistent cohorts within and across trials when using different Aβ tracers. In the moderate CL range (25–50CL), typically associated with moderate plaques and negative tau PET,^34^ all NAV scans (*n* = 6) were read as positive by both reader groups, while 71.4% of FBP scans (5 out of 7) were read as negative. In the 50–100CL range, where plaques are more frequent, 5.9% of FBP scans were still read as negative, whereas all NAV scans were read as positive.

Our findings also support previous reports that the standard whole cerebellum reference region is not optimal for CL quantification of FBP and the addition of hemispheric white matter to the reference region leads to a better match of the FBP CL to both visual read results and to the same subject NAV CL.

Limitations: We used CL quantification as the reference standard due to its widespread clinical and research use. However, imperfections and outliers in quantified data may cause discordance with visual reads, highlighting the need for advanced techniques to improve tracer quantification. Data were acquired using different scanners and PET reconstruction methods. As this is inevitable for multi‐center studies, better harmonization approaches could minimize scanner‐specific effects and improve quantification output.^35^ Readers were restricted from using structural imaging (computed tomography/magnetic resonance imaging [CT/MRI]), typically available in clinical practice. Incorporating anatomical images alongside PET could improve visual performance, especially in challenging cases with severe brain atrophy.

Another limitation of this study is the use of different standard color scales (rainbow for NAV and inverted grayscale for FBP) in visual reading. Color scale choice may influence perception of gray‐white matter contrast and visual read sensitivity, particularly at low or borderline amyloid burden. A supplementary analysis in a subset of participants (*n* = 20), including all discordant cases from the primary analysis (*n *= 9) and five positive and six negative cases (Figure S5 and S6, Table S4), showed that using rainbow color scale for FBP reduced overall visual discordance from 50% (10 NAV+/FBP‐, 0 NAV‐/FBP+) to 35% (6 NAV+/FBP‐, 1 NAV‐/FBP+). This suggests a modest gain in sensitivity in some discordant cases, although borderline positives may still be missed compared with NAV (Figure S7). Notably, use of alternative color scales also led to a visually negative, CL– case being classified as positive, indicating a potential increase in false positives. These findings suggest that color scale selection can affect both sensitivity and specificity and warrant systematic evaluation across tracers and reader experience levels in future studies.

The dataset included a near‐equal split of positive and negative cases based on the FBP 20CL cutoff, reflecting a typical memory clinic scenario. However, most cognitively unimpaired participants were between ‐10 and 5CL, while most cognitively impaired were above 60 CL, limiting the sample size between 10 and 70CL. Consequently, most scans were clearly positive or negative, so both tracers achieved high overall accuracy despite variability in the small group with low to moderate Aβ. A larger head‐to‐head study focused on this low to moderate Aβ range would strengthen confidence in our findings.^17^ Ideally, scans acquired within weeks would eliminate bias from amyloid accumulation, which occurs at ∼3–4CL per year.^36^ In this study, most participants had FBP before NAV, but this likely had minimal impact: CU participants showed identical CL values; NAV was only 3CL higher in MCI with an average 10‐month gap and 5CL higher in AD with a 6‐month gap.

In summary, with accuracy, sensitivity, and specificity above 90% at a lower CL threshold, the visual reader performance with NAV was more robust compared to FBP. Moreover, both experienced and novice readers performed equally well with NAV, having comparable performance metrics and inter‐reader agreement scores at similar CL thresholds. Our findings support the use of Aβ NAV for the detection of low levels of cerebral Aβ plaques more accurately than FBP, aiding the visual exclusion of low to moderate levels of Aβ pathology.

## AUTHOR CONTRIBUTIONS

Ishara Paranawithana, Vincent Doré, and Christopher C. Rowe contributed to the study concept and design, formal analysis of data, visualization, and writing the original draft. Victor L. Villemagne, Ashley G. Gillman, and Azadeh Feizpour contributed to methodology. Christopher C. Rowe, Aurora Poon, H. B. Toh, Tanyaluck Thientunyakit, Tawika Kaewchur, and Antony Sutherland contributed to visual read of Aβ PET scans. Pierrick Bourgeat, Vincent Doré, and Kun Huang contributed to data curation. Christopher C. Rowe, and Jurgen Fripp contributed to funding acquisition. Vincent Doré and Pierrick Bourgeat contributed to statistical review. Vincent Doré, Pierrick Bourgeat, Christopher C. Rowe, and Victor L. Villemagne contributed to review and editing. All authors contributed to critical review of the manuscript, provided final approval of the version to be published, and accepted responsibility for the accuracy and integrity of the reported work.

## CONFLICT OF INTEREST STATEMENT

C.C.R. has received research grants from NHMRC Australia, Medical Research Future Fund, Enigma Australia, Biogen, Eisai, Roche, and Abbvie. He is on the scientific advisory board for Enigma Biomedical Group and Prothena, Roche, Eisai, Eisai Australia, Eli Lilly Australia, Novo Nordisk Australia. V.L.V. has been a consultant or paid speaker at sponsored conference sessions for Eli Lilly and Company, Life Molecular Imaging, ACE Barcelona, and Lantheus. The other authors did not report any conflict of interest. Author disclosures are available in the Supporting Information.

## CONSENT STATEMENT

The study was approved by the human research ethics committee at St. Vincent's Hospital, Melbourne (HREC 028/06). Written informed consent was obtained from all subjects or the next of kin/caregiver for people living with dementia before participation in the study.

## Supporting information




**Supporting Information**: dad270426‐supp‐0001‐SupMat
