# Supplementary material for: Visual read performance of 18F‐Florbetapir and 18F‐NAV4694 Aβ PET compared against Centiloid reference standard in a paired cohort
Source: Alzheimers Dement (Amst). 2026 Jul 16;18(3):e70426. doi: 10.1002/dad2.70426 (PMC13375940; doi:10.1002/dad2.70426)
Supplement: Supplementary file 1 — Supporting Information: dad270426‐supp‐0001‐SupMat [file DAD2-18-e70426-s001.docx]

# Supplementary Figures/Tables

| 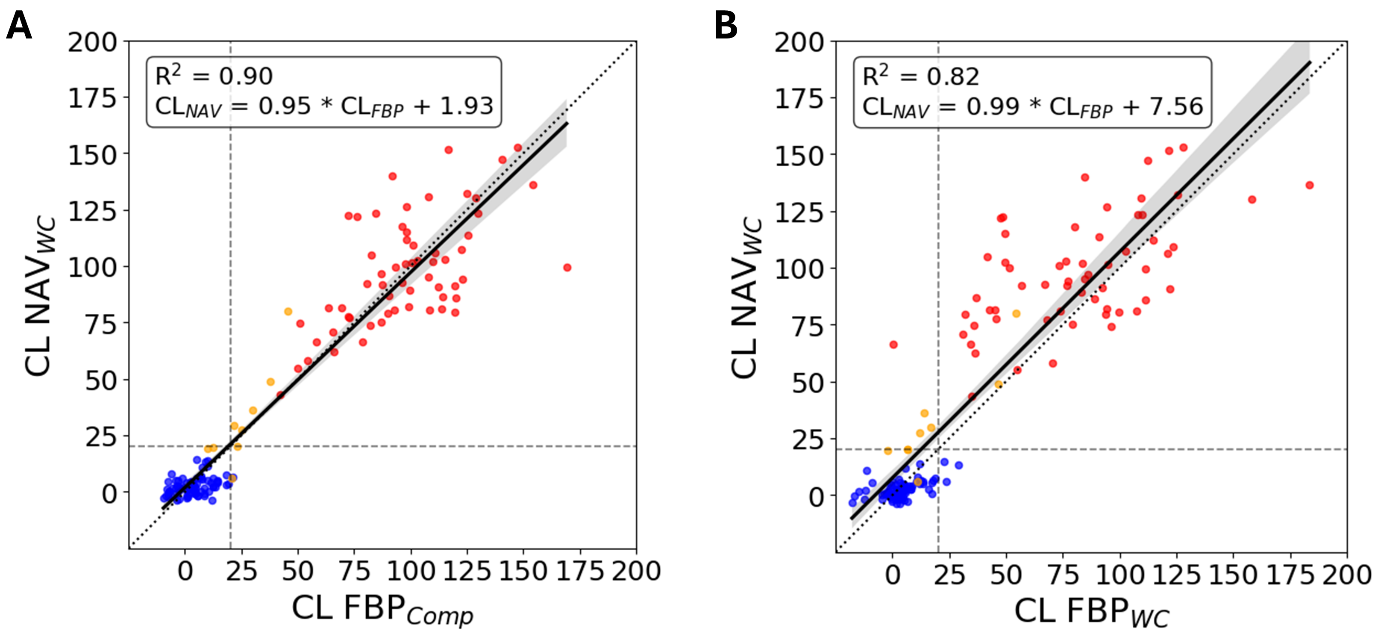 |
| --- |
| Figure S1: Comparison of CL quantification agreement between NAV and FBP with different reference regions; (A) composite region comprising subcortical white matter and the whole cerebellum and (B) whole cerebellum. Datapoints are color-coded based on the majority vote of visual readers (>=4) with both NAV and FBP positive in red, both NAV and FBP negative in blue and NAV positive, FBP negative in yellow. Abbreviations: WC: whole cerebellum, Comp: composite white matter. |

| 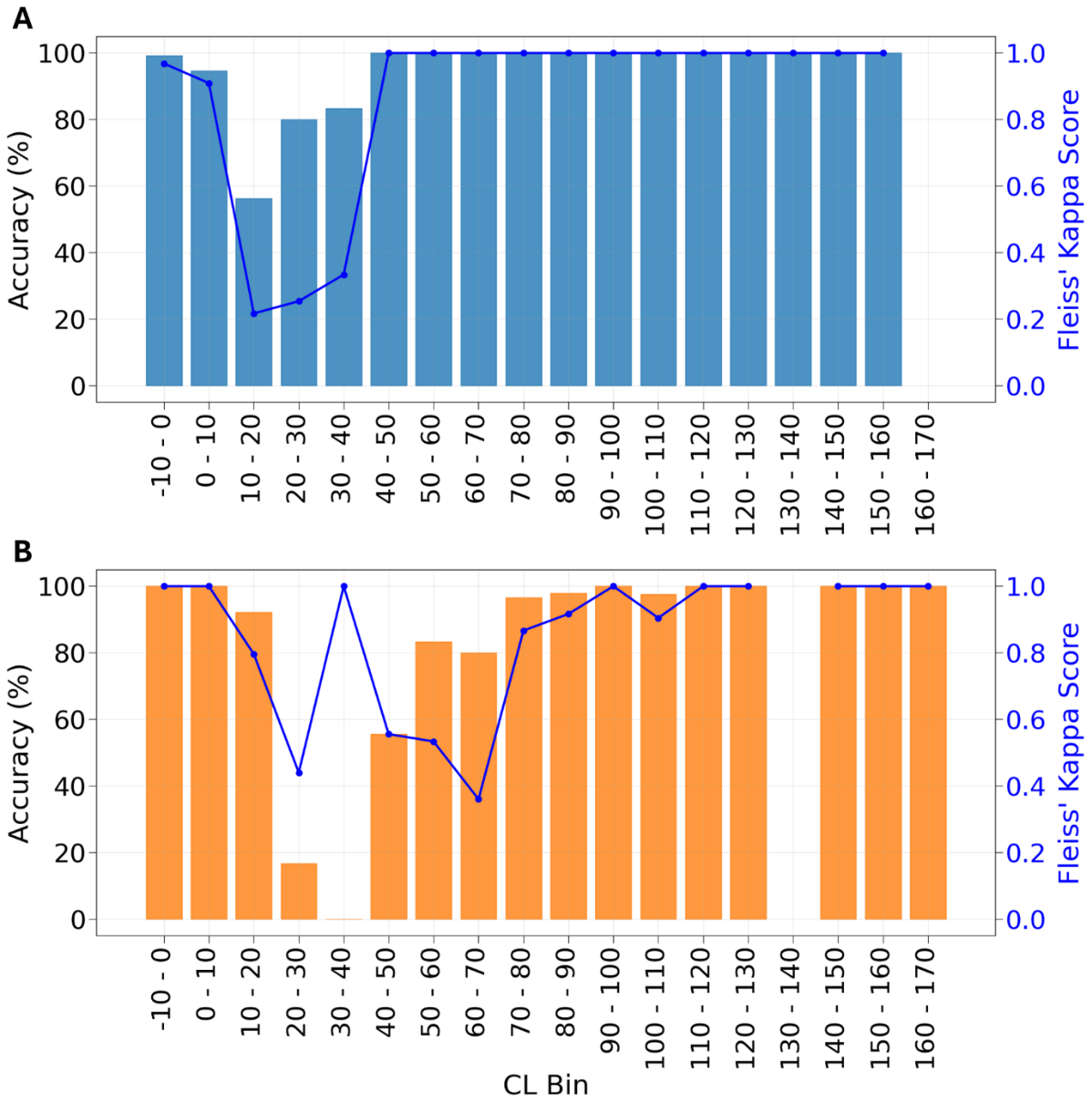 |
| --- |
| Figure S2: Combined reader (expert plus novice) accuracy of visual assessment (mean and 95% confidence intervals shown in bar charts) and inter-reader agreement measured by Fleiss’ Kappa scores (in line plots) for (A) $\text{NAV}_{\text{WC}}$ and (B) $\text{FBP}_{\text{Comp}}$ for the entire Centiloid range with a bin size of 10CL. Note that a generally accepted threshold of 20CL was used for both tracers in the accuracy analysis. |

| 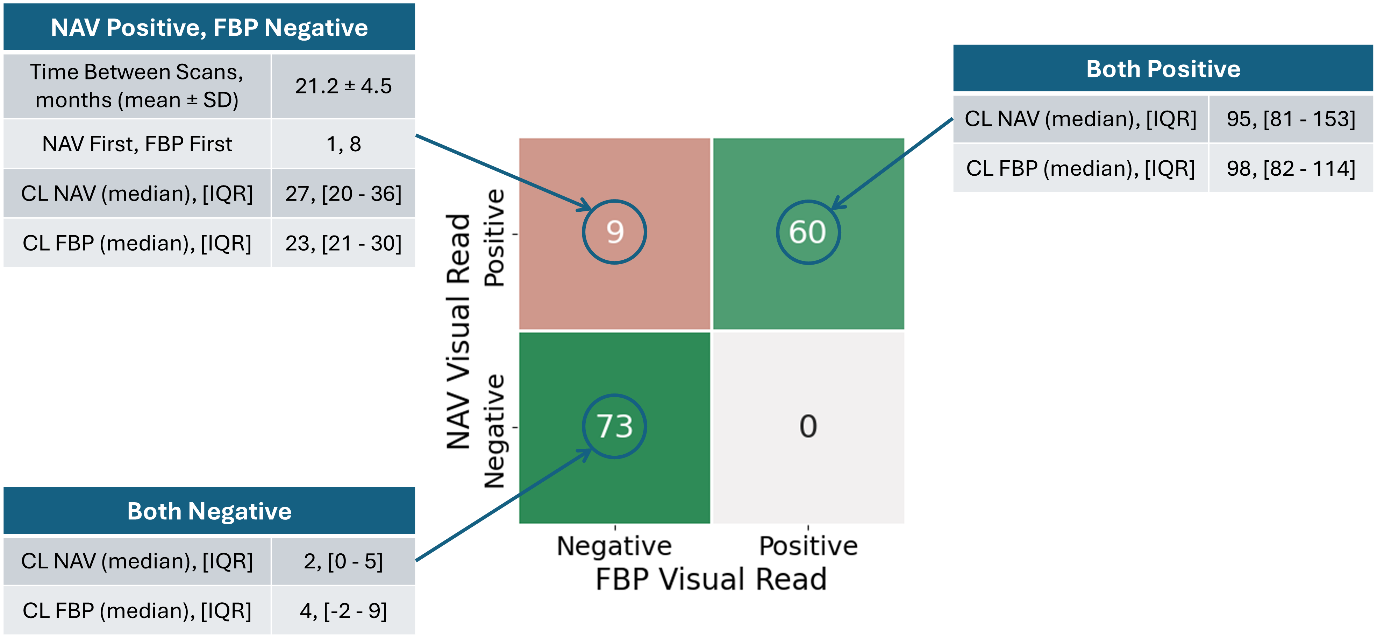 |
| --- |
| Figure S4: Comparison of NAV and FBP visual read agreement based on the majority vote (>=4 readers). The confusion matrix shows that most cases were concordant (NAV positive and FBP positive: 60, NAV negative and FBP negative: 73). However, a small number of discordant cases exists (NAV positive and FBP negative: 9). The median and range of CL values are provided for each case and the average time difference between scans and the order of scans are also reported for discordant cases. |

| 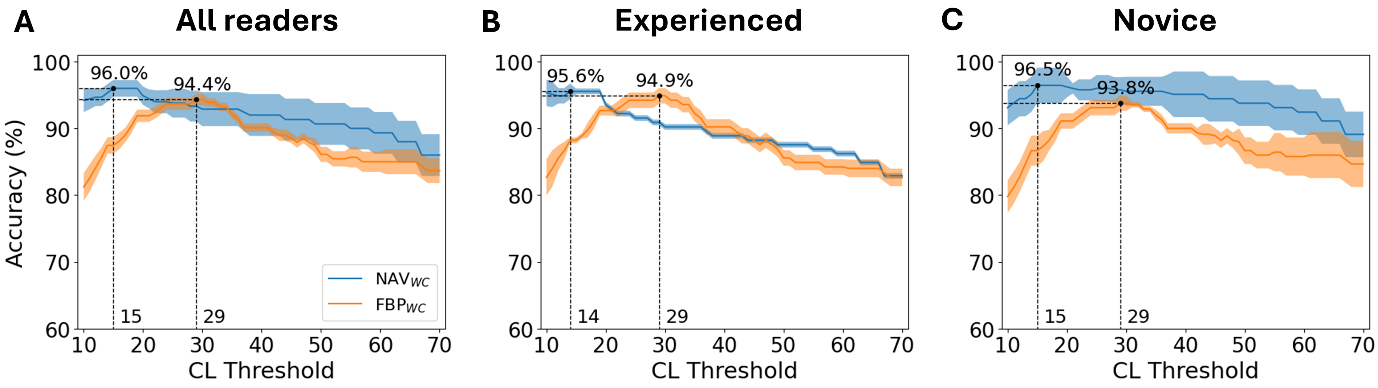 |
| --- |
| Figure S3: Accuracy of visual assessment of Aβ PET scans against CL reference standard (using whole cerebellum as the reference region for both tracers) for thresholds between 10 and 70CL for (A) all readers combined, (B) experienced readers and (C) novice readers. The peak accuracy and corresponding threshold value for each tracer is annotated in each plot. Error bars represent 95% CI across readers. |

| 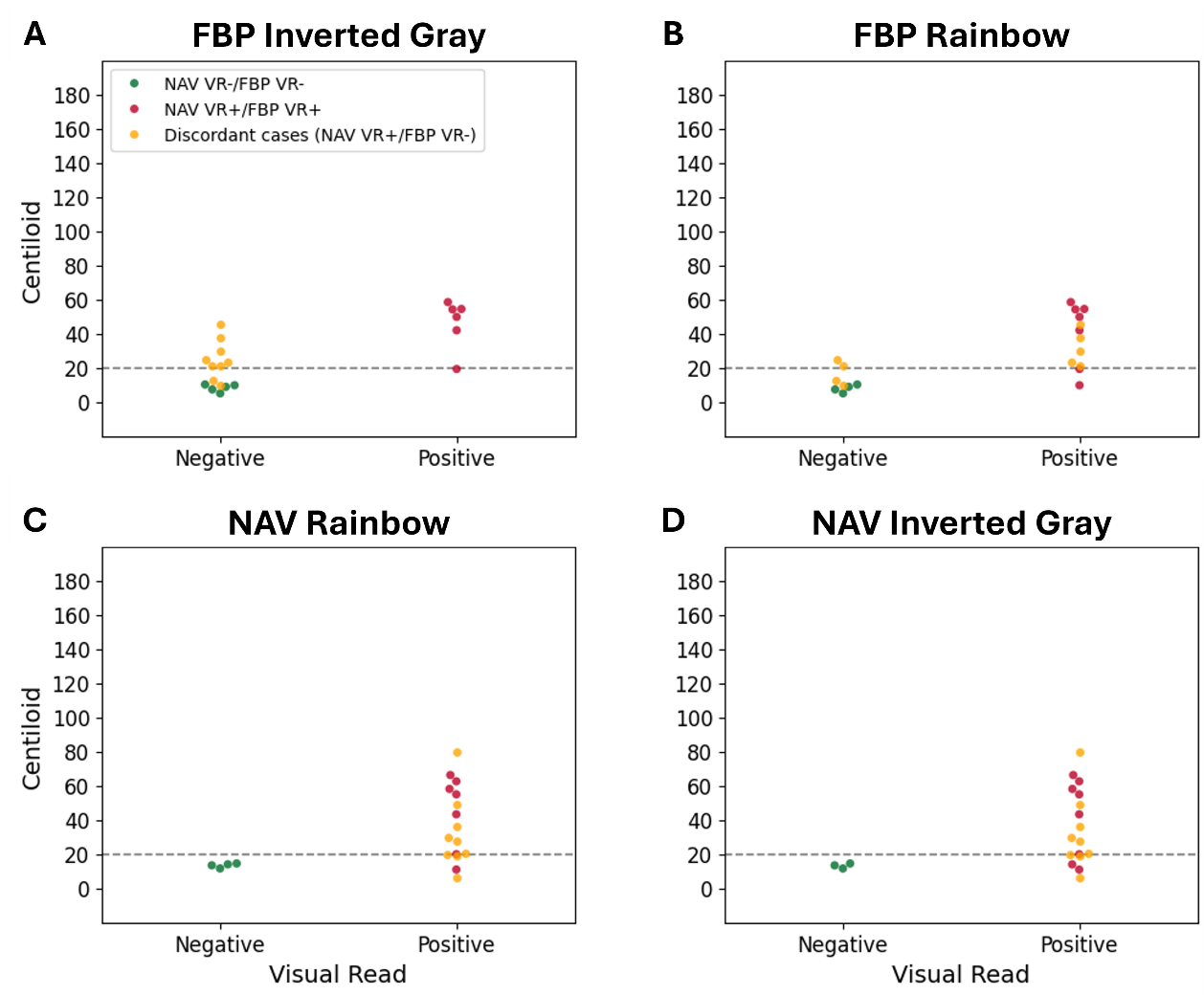 |
| --- |
| Figure S5: Swarm plots of CL versus visual read results of an expert reader (A.P.) from supplementary analysis conducted for a subset of participants (n=20) with all 9 discordant cases (visual read NAV+/FBP- from the primary analysis marked in yellow) and another 5 positive and 6 negative cases based on FBP CL. The left and right columns represent standard color scale and alternative color scale, respectively. The swarm plots depict results for (A) FBP in inverted grayscale, (B) FBP in rainbow color scale, (C) NAV in rainbow color scale and (D) NAV in inverted grayscale. |

| 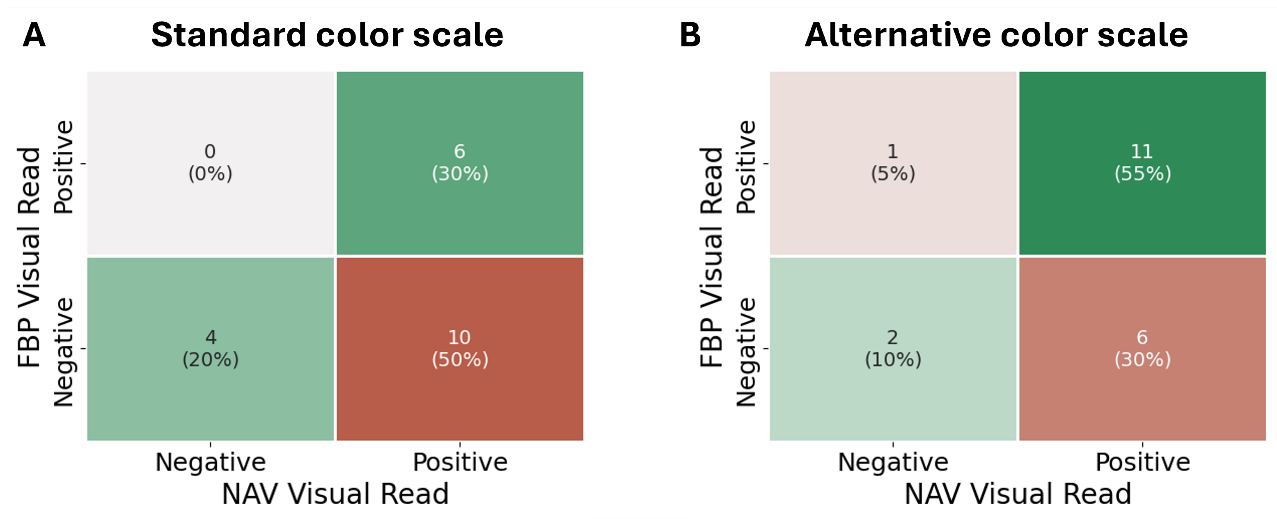 |
| --- |
| Figure S6: Comparison of visual read agreement between NAV and FBP when using (A) standard and (B) alternative color scales. Results from the supplementary analysis (n=20) show that using rainbow color scale for FBP reduced overall visual discordance from 50% (10 NAV+/FBP-, 0 NAV-/FBP+) to 35% (6 NAV+/FBP-, 1 NAV-/FBP+). |

| 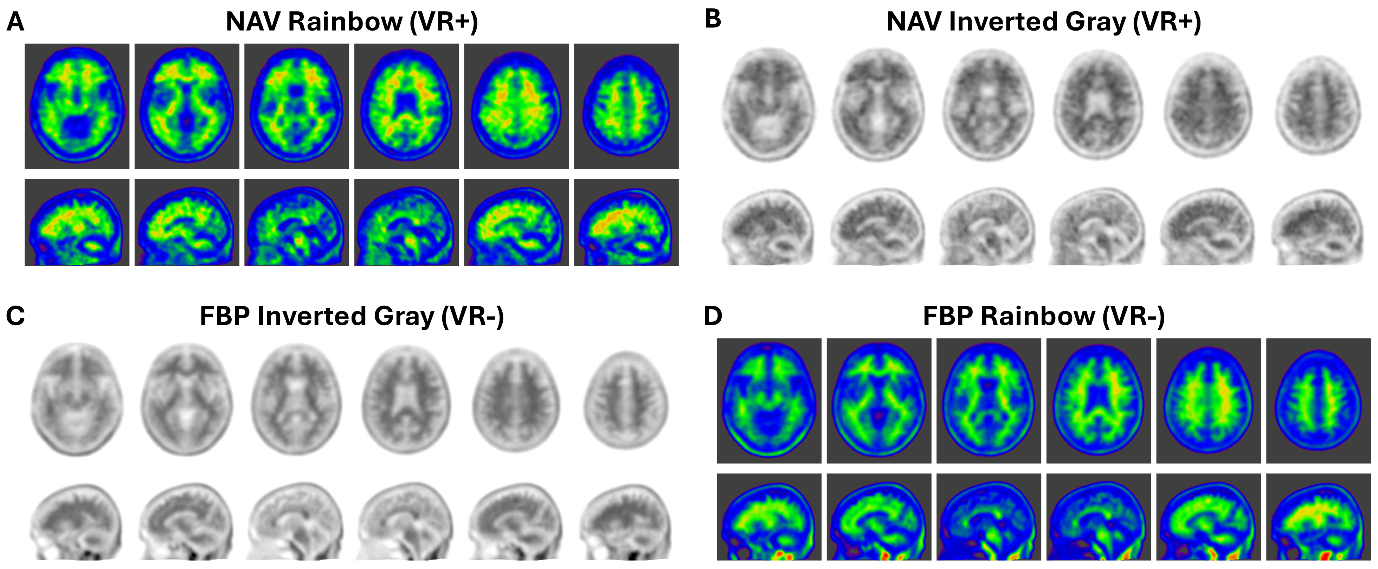 |
| --- |
| Figure S7: Comparison of PET images of a sample case where visual read by an expert reader (A.P.) was NAV+/FBP- with standard color scales and remained FBP- with the alternative rainbow color scale. The left and right columns represent standard (rainbow for NAV and inverted gray for FBP) and alternative color scales, respectively. NAV images are shown in the top row and FBP images are shown in the bottom row. Six transaxial and sagittal slices of PET images are presented for each tracer. The global amyloid burden estimated with CapAIBL was 27.4CL for NAV and 25.1CL for FBP. Five readers (all 3 experts and 2 novices) had read the NAV scan as positive, and all readers had read FBP scan as negative in standard color scales. |

| Table S1: Participant demographics and characteristics of different clinical groups | | | | |
| --- | --- | --- | --- | --- |
|  | **CU** | **MCI** | **AD** | **Total** |
| **Sample size** | 88 | 40 | 22 | 150 |
| **Scan order (NAV first, FBP first)** | 3, 85 | 35, 5 | 21, 1 | 59, 91 |
| **Age (years), mean ± SD** | 71.9 ± 4.3 | 71.8 ± 7.1 | 68.0 ± 8.5^*^ | 71.3 ± 6.0 |
| **Gender (male), N (%)** | 44 (50.0%) | 24 (60.0%) | 7 (31.8%) | 75 (50.0%) |
| **MMSE, median (IQR)** | 29.0 (2.0)^†^ | 25.0 (3.0)^*^ | 23.0 (3.5)^*†^ | 28.0 (4.0) |
| **CDR-SoB, median (IQR)** | 0.0 (0.0)^†^ | 2.0 (1.1)^*^ | 4.0 (0.5)^*†^ | 0.0 (2.0) |
| **APOE ε4+, N (%)** | 25 (28.7%)^†^ | 33 (84.6%)^*^ | 14 (66.7%)^*^ | 72 (49.0%) |
| **NAV CL, mean ± SD** | 8.4 ± 16.7^†^ | 88.8 ± 30.8^*^ | 104.6 ± 25.3^*†^ | 44.0 ± 48.3 |
| **FBP CL, mean ± SD** | 8.7 ± 15.2^†^ | 91.6 ± 32.6^*^ | 99.5 ± 26.6^*^ | 44.1 ± 48.1 |
| **Interval between scans (months), mean ± SD** | 21.2 ± 3.3^†^ | 9.9 ± 5.1^*^ | 6.4 ± 2.7^*†^ | 16.0 ± 7.3 |
| Abbreviations: CU - cognitively unimpaired, MCI - mild cognitive impairment, AD - Alzheimer’s disease, N - number of individuals, MMSE - Mini-Mental State Examination, CDR-SoB - Clinical Dementia Rating Scale Sum of Boxes, ^*^p-value < 0.05 compared to CU. ^†^p-value < 0.05 compared to MCI. SD and IQR refer to the standard deviation and inter-quartile range, respectively. | | | | |

| Table S2: Comparison of visual read performance of **experienced** readers in (A) reader 1, (B) reader 2 and (C) reader 3 for NAV an FBP tracers against Centiloid quantification (threshold: 20CL) | | | | | | | | | | | | | | | | | |
| --- | --- | --- | --- | --- | --- | --- | --- | --- | --- | --- | --- | --- | --- | --- | --- | --- | --- |
| **A** | **NAV** | | | | | | | |  | | **FBP** | | | | | | |
|  |  | | CL quantification | | | | | |  | |  | | CL quantification | | | | |
|  | Visual read | | Positive | | Negative | | Total | |  | | Visual read | | Positive | | Negative | | Total |
|  | Positive | | 71 | | 10 | | 81 | |  | | Positive | | 67 | | 1 | | 68 |
|  | Negative | | 0 | | 69 | | 69 | |  | | Negative | | 7 | | 75 | | 82 |
|  | Total | | 71 | | 79 | | 150 | |  | | Total | | 74 | | 76 | | 150 |
| **B** |  |  | |  | |  | |  | |  | |  | |  | |  | |
|  | Positive | | 71 | | 9 | | 80 | |  | | Positive | | 63 | | 2 | | 65 |
|  | Negative | | 0 | | 70 | | 70 | |  | | Negative | | 11 | | 74 | | 85 |
|  | Total | | 71 | | 79 | | 150 | |  | | Total | | 74 | | 76 | | 150 |
| **C** |  |  | |  | |  | |  | |  | |  | |  | |  | |
|  | Positive | | 71 | | 10 | | 81 | |  | | Positive | | 61 | | 2 | | 63 |
|  | Negative | | 0 | | 69 | | 69 | |  | | Negative | | 13 | | 74 | | 87 |
|  | Total | | 71 | | 79 | | 150 | |  | | Total | | 74 | | 76 | | 150 |

| Table S3: Comparison of visual read performance of **novice** readers in (A) reader 4, (B) reader 5 and (C) reader 6 for NAV an FBP tracers against Centiloid quantification (threshold: 20CL) | | | | | | | | | | | | | | | | | |
| --- | --- | --- | --- | --- | --- | --- | --- | --- | --- | --- | --- | --- | --- | --- | --- | --- | --- |
| **A** | **NAV** | | | | | | | |  | | **FBP** | | | | | | |
|  |  | | CL quantification | | | | | |  | |  | | CL quantification | | | | |
|  | Visual read | | Positive | | Negative | | Total | |  | | Visual read | | Positive | | Negative | | Total |
|  | Positive | | 69 | | 2 | | 71 | |  | | Positive | | 61 | | 2 | | 63 |
|  | Negative | | 2 | | 77 | | 79 | |  | | Negative | | 13 | | 74 | | 87 |
|  | Total | | 71 | | 79 | | 150 | |  | | Total | | 74 | | 76 | | 150 |
| **B** |  |  | |  | |  | |  | |  | |  | |  | |  | |
|  | Positive | | 65 | | 2 | | 67 | |  | | Positive | | 54 | | 0 | | 54 |
|  | Negative | | 6 | | 77 | | 83 | |  | | Negative | | 20 | | 76 | | 96 |
|  | Total | | 71 | | 79 | | 150 | |  | | Total | | 74 | | 76 | | 150 |
| **C** |  |  | |  | |  | |  | |  | |  | |  | |  | |
|  | Positive | | 71 | | 5 | | 76 | |  | | Positive | | 61 | | 1 | | 62 |
|  | Negative | | 0 | | 74 | | 74 | |  | | Negative | | 13 | | 75 | | 88 |
|  | Total | | 71 | | 79 | | 150 | |  | | Total | | 74 | | 76 | | 150 |

| Table S4: Summary of FBP visual read results of visually discordant cases (NAV+/FBP-) in standard (inverted gray) and alternative (rainbow) color scales. Reader’s confidence rating on a scale of 0-100% is reported for each color scale. The case study presented in Figure S7 is highlighted in yellow. | | | | | |
| --- | --- | --- | --- | --- | --- |
|  | Standard (Inverted Grayscale) | | Alternative (Rainbow) | | CL |
|  | VR | Reader's Confidence  (0-100%) | VR | Reader's Confidence  (0-100%) |  |
| Case 1 | Negative | 70 | Positive | 80 | 45.7 |
| Case 2 | Negative | 90 | Positive | 70 | 37.9 |
| Case 3 | Negative | 90 | Positive | 70 | 29.9 |
| Case 4 | Negative | 100 | Positive | 90 | 23.2 |
| Case 5 | Negative | 90 | Positive | 90 | 20.1 |
| Mean (SD) |  | 88.0 (11.0) |  | 80.0 (10.0) | 31.4 (10.5) |
|  | | | | | |
| Case 6 | Negative | 100 | Negative | 70 | 25.1 |
| Case 7 | Negative | 100 | Negative | 80 | 21.5 |
| Case 8 | Negative | 100 | Negative | 50 | 12.5 |
| Case 9 | Negative | 90 | Negative | 80 | 9.8 |
| Mean (SD) |  | 97.5 (5.0) |  | 70.0 (14.1) | 17.2 (7.3) |
